# Delivery after Operation for Deeply Infiltrating Endometriosis 

**DOI:** 10.1155/2016/8271452

**Published:** 2016-07-19

**Authors:** Christina Allerstorfer, Peter Oppelt, Simon H. Enzelsberger, Andreas Shamiyeh, Wolfgang Schimetta, Omar Josef Shebl, Richard Bernhard Mayer

**Affiliations:** ^1^Department of Obstetrics and Gynecology, Kepler University Clinic, Campus IV, Krankenhausstrasse 26-30, 4020 Linz, Austria; ^2^Second Surgical Department, Ludwig Boltzmann Institute for Surgical Laparoscopy, Kepler University Clinic, Campus III, Krankenhausstrasse 9, 4020 Linz, Austria; ^3^Department of Applied Systems Research and Statistics, Johannes Kepler University, 4040 Linz, Austria

## Abstract

*Background.* It has been suggested that, during pregnancy, endometriosis can cause a variety of disease-related complications.* Objectives.* The purpose of the study was to find out if women with histologically confirmed endometriosis do have a higher risk of adverse pregnancy outcome and if they suffer from a higher rate of complications during labor.* Study Design.* 51 women who underwent surgery because of deeply infiltrating endometriosis in the General Hospital Linz and the Women's General Hospital Linz and who gave birth in the Women's General Hospital Linz after the surgery were included in our survey.* Results.* 31 women (60.8%) had a spontaneous delivery and in 20 women (39.2%) a caesarean section was performed. There were no cases of third- and fourth-degree perineal lacerations. Collectively there were 4 cases (7.8%) of preterm delivery and one case (2.0%) of premature rupture of membranes. In two women (6.5%) a retained placenta was diagnosed.* Conclusions.* Our study is the first description on delivery modes after surgery for deeply infiltrating endometriosis. We did not find an elevated risk for perineal or vaginal laceration in women with a history of surgery for deeply infiltrating endometriosis, even when a resection of the rectum or of the posterior vaginal wall had been performed.

## 1. Introduction

Endometriosis is defined as the presence of endometrium-like tissue outside of the uterine cavity [1]. Deeply infiltrating endometriosis (DIE) is defined as endometriotic lesions in the rectovaginal septum, the vaginal fornix, and the peritoneum or if the bowel, the ureter, or the bladder is infiltrated by the disease [2].

Due to the lack of large cohort studies, the prevalence of endometriosis is still unknown [3], but it is estimated that 10 to 15 percent of women in the reproductive age are affected by the disease [4]. As endometriosis is regarded to be a condition of the premenopausal woman, its peak of prevalence coincides with a woman's reproductive period [5].

The question if endometriosis has an influence on fertility, pregnancy, and obstetrical outcome has been subject to different studies in the last years.

It has been suggested that, during pregnancy, endometriosis can cause a variety of disease-related complications, such as miscarriage, bleeding from ectopic implants, preterm birth, fetal growth restriction, preeclampsia, or obstetrical bleeding [6].

Endometriosis is regarded as the major risk factor for SHiP (sudden hemoperitoneum in pregnancy), which is a rare but potentially hazardous complication that can be caused by the decidualization of ectopic lesions during pregnancy and in the postpartum period [3, 7]. But not only can decidualization cause massive intraperitoneal hemorrhage, it can also lead to weakening of the intestinal wall and therefore cause spontaneous rectal perforation during pregnancy [8, 9]. So far 12 cases of bowel perforation during pregnancy have been reported, but endometriosis has been diagnosed in 3 cases only [6].

The influence of endometriosis on the development of preeclampsia is debatable. Brosens et al. showed a significantly reduced risk of preeclampsia in women with endometriosis, while Falconer reported that women with endometriosis seem to be at a higher risk for the development of preeclampsia [10, 11].

The risk of preterm birth seems to be elevated in women with endometriosis, and Conti et al. also found an increased risk for SGA fetuses and preterm premature rupture of membranes [12–14].

Women with endometriosis seem to be at higher risk for placenta previa, especially when there are rectovaginal endometriotic lesions present [15]. According to a study from Vercellini et al. the rate of placenta previa in women with endometriosis is more than tenfold higher than in the general population (3.7 percent versus 0.3 percent) [15].

While possible influences of endometriosis on pregnancy have been described, little is known about the influence the disease can have on the obstetrical outcome. While two studies described a higher risk for delivery through caesarean section in women with endometriosis, a study performed by Conti et al. did not find a positive correlation [3, 13, 14].

There are no guidelines concerning the mode of delivery in pregnant women after surgery for deeply infiltrating endometriosis. Even the “Guideline on the Management of Women with Endometriosis” does not address this issue [16]. The “Guideline for Diagnosis and Therapy of Endometriosis” only suggests that the mode of delivery should be discussed with each patient individually [17].

*Hypothesis.* Do women with histologically confirmed endometriosis have a higher risk of adverse pregnancy outcome or complications during delivery and does the diagnosis of deeply infiltrating endometriosis (DIE) have influence on the choice of the delivery mode?

## 2. Materials and Methods

We included all women on whom surgery because of deeply infiltrating endometriosis was performed in the General Hospital Linz between 01.01.2009 and 31.12.2013 and in the Women's General Hospital Linz between 01.01.2013 and 31.12.2013 and who gave birth in the Women's General Hospital Linz after the surgery until 31.03.2015 (the General Hospital Linz and the Women's General Hospital Linz are now part of the Kepler University Clinic). Only women with histologically verified endometriotic lesions were included in the survey.

We looked through the patient records to gain information about the surgery as well as details about the delivery.

Concerning the performed surgery we collected the following information:

- Date of surgery.
- Performed surgery.
- ENZIAN classification.
- rAFS classification.
- Affected structures (adenomyosis, endometriosis of the ovary, endometriosis of the fallopian tube, endometriosis of the peritoneum, endometriosis of the vagina, endometriosis of the rectovaginal septum, endometriosis of the uterovesical fold, endometriosis of the colon, and endometriosis of the ureter).
- Duration of surgery.

**Figure 1:**
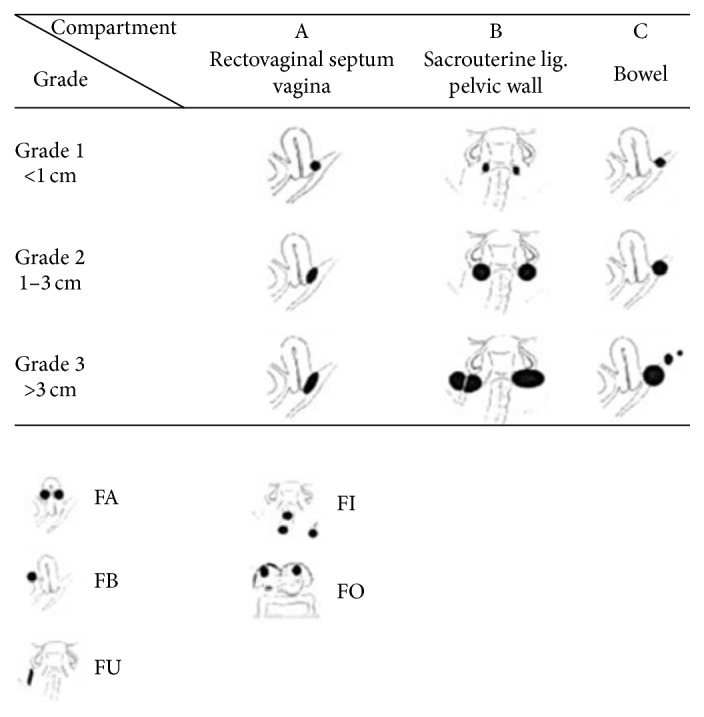
The revised ENZIAN classification of endometriosis.

We used the ENZIAN classification to classify all patients because it is a common and validated classification system for deeply infiltrating endometriosis (Figure 1) [18].

Concerning the delivery we extracted the following information from the patient records:

- Number of previous pregnancies.
- Number of previous births.
- Period of time between the surgery and the pregnancy.
- Artificial reproduction techniques.
- Duration of pregnancy.
- Complications during pregnancy (gestational diabetes, partus praematurus, premature rupture of membranes, placenta previa, preeclampsia, placental abruption, and placental retention).
- Mode of delivery.
- Duration of expulsion stage.
- Birth injuries.
- Length of stay in the hospital.
- Data of the newborn (birth weight, body length, head circumference, APGAR score, and umbilical blood gases).

Two-sided 95% confidence intervals (95% CI) were calculated for the incidences of several kinds of birth procedures and complications as well as for the duration of the expulsion stage.

For subgroup comparisons (spontaneous delivery versus caesarean) all data sets of metric variables were checked for normal distribution (test of normality: Kolmogorov-Smirnov with Lilliefors significance correction, type I error = 10%).

Normally distributed data sets were compared by the *t*-test (test for variance homogeneity: Levene test, type I error = 5%) for independent samples, metric variables without normally distributed data sets and variables measured on ordinal scales by the exact Mann-Whitney *U* test, and dichotomous variables by Fisher's exact test.

Logistic regression analysis (forward stepwise method with Wald statistics) and multiple regression analysis (stepwise) were carried out to detect independent prognostic factors for caesarean and for the duration of the expulsion stage.

Independent variables were as follows:

- ENZIAN A.
- ENZIAN B.
- ENZIAN C.
- Age at the time of surgery (years).
- Period of time between surgery and delivery (months).
- Duration of surgery (minutes).
- Number of parturition processes (before the surgery).
- Birth weight (gram).
- Head circumference (centimeter).

Type I error was not adjusted for multiple testing. Therefore the results of inferential statistics are descriptive only. Statistical analysis was performed using the open-source *R* statistical software package, version 3.0.2.

Approval for the survey was obtained by the local institutional ethics committee on 20 July 2015 (reference number K-71-15).

## 3. Results

A total of 51 women met all inclusion criteria and were included in our survey.

The average age at the time of surgery was 29.2 years ± 4.27 (mean ± SD).

In 12 cases the left rectouterine ligament was removed, in 7 cases the right rectouterine ligament was removed, and in 8 patients the rectouterine ligament had to be removed on both sides. In three patients, the vagina was surgically opened because endometriotic lesions in this area were removed. A resection of the sigmoid colon was performed in one case, and in three patients a resection of the rectum was carried out.

**Figure 2:**
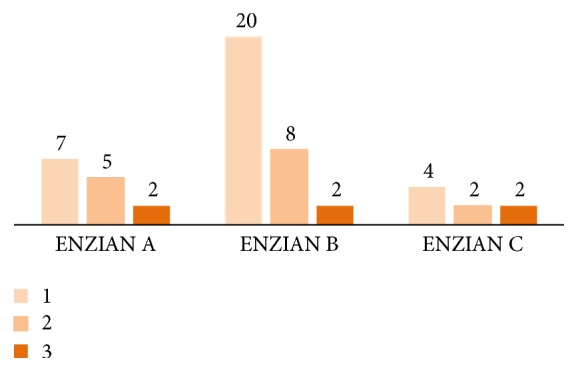
Patients described by the ENZIAN classification.

In 14 patients, the vagina or the rectovaginal septum was affected by endometriosis (ENZIAN A), in 30 cases the rectouterine ligaments, the parametria, the pelvic wall, or the ureter (extrinsically) was affected (ENZIAN B), and in 8 patients infiltration of the rectum (ENZIAN C) was diagnosed (Figure 2).

Concerning the mode of delivery, 31 women (60.8%) had a spontaneous delivery and in 20 women (39.2%) a caesarean section was performed: in 11 of these 20 cases a primary caesarean section was performed, and in 4 cases the indication for the primary caesarean section was the endometriosis itself. In 9 cases a secondary caesarean section had to be performed.

The mean period of time between the surgery and the delivery was 19.7 months in the group of women that had a caesarean section and 22.7 months in the group of women that gave birth spontaneously.

In the group of spontaneous births there were 7 cases (22.6%) of first-degree perineal tears and 6 cases (19.4%) of second-degree perineal tears. Collectively there were no cases of third- or fourth-degree perineal tears. An episiotomy was performed in 10 cases (32.3%). In three women (9.7%) a vaginal tear was diagnosed.

There were 4 cases (7.8%) of preterm delivery and one case (2.0%) of premature rupture of membranes. In two women (6.5%) a manual removal of the placenta because of a retained placenta had to be performed.

Collectively there were no cases of placenta previa or preeclampsia.

When looking at the umbilical cord gas the mean arterial pH was 7.30 when a caesarean section had been performed and 7.27 in women who gave birth spontaneously, which means that, concerning the umbilical cord cases, there was no statistically significant difference between these two groups (*p* = 0.054) (Table 1).

Table 2 shows the obstetrical outcome for women in which surgery in compartment ENZIAN A had been performed. Five of these 14 women gave birth spontaneously and there was no case of vaginal laceration. In 9 cases a caesarean section was performed (5 cases of primary caesarean section and 4 cases of secondary caesarean section). In 4 of the 5 cases of primary caesarean section, the indication for the caesarean operation was provided because of the endometriosis.

Table 3 shows information about the delivery mode in women with surgery in the compartment ENZIAN C. Of the affected 8 women, only two gave birth spontaneously. In 5 cases a primary caesarean section and in one case a secondary caesarean section were performed. In 4 of the 5 cases of primary caesarean operation, the caesarean section was performed because of the endometriosis.

One of the two women that had a spontaneous delivery had a history of rectum resection due to endometriosis and there was no fourth-degree perineal laceration in this woman.

Our results show that operation for deeply infiltrating endometriosis does have a statistically significant influence on the choice of the delivery mode when the compartments ENZIAN A and C are affected or a rectum resection has been performed (Table 4).

There was no higher risk for pregnancy complications or adverse pregnancy outcome collectively.

## 4. Comments

Previous studies have shown that deeply infiltrating endometriosis can be associated with a various number of complications during pregnancy and delivery [6].

However, our results did not show an association between previous surgery due to deeply infiltrating endometriosis and adverse pregnancy outcome.

Our results do show that the risk for abdominal delivery seems to be elevated in women with endometriosis. The caesarean rate collectively was 39.2%, while according to Statistik Austria in 2014 the rate of abdominal delivery in the overall population in Austria was 29.8% [19]. This finding is conclusive with the results of a survey conducted by Stephansson et al., who also found a highly elevated rate of caesarean sections in women with endometriosis compared to women without the disease [12].

Collectively, women with endometriosis of the compartment ENZIAN A or ENZIAN C had a statistically higher risk for delivery through caesarean section than women without endometriosis in these compartments (*p* = 0.020 and *p* = 0.031). There was also a statistically significant elevated caesarean rate when a rectal resection had been performed (*p* = 0.029).

The mean period of time between the surgery and the delivery was longer in the group of women that gave birth spontaneously than in the group of women where a caesarean section was performed (22.7 months versus 19.7 months), although this finding was not statistically significant (*p* = 0.329).

The influence of endometriosis on the development of preeclampsia during pregnancy is still unclear. Brosens et al. found that the risk for preeclampsia in women with endometriosis can be increased, decreased, or unchanged [3]. Collectively, we did not have a case of preeclampsia.

Although Lin et al. showed an increased risk of placenta previa in pregnant women with endometriosis [14], we cannot confirm this observation as there was no case of placenta previa collectively. We also found no case of placental abruption.

There are case reports of massive gastrointestinal or intraperitoneal bleedings due to decidualization of endometriotic lesions during pregnancy or in the postpartum period [3, 7]. However, collectively we did not have a complication like these.

Menzlova et al. reported a case about a fourth-degree perineal laceration after spontaneous birth in a woman with previously diagnosed endometriosis of the rectovaginal septum [20]. Collectively, endometriosis of the compartment ENZIAN A was diagnosed in 14 women, of whom 5 women gave birth spontaneously. One of these five women even had a history of surgical opening of the vagina because endometriotic lesions in the vaginal wall had to be removed. However, we had no case of third- or fourth-degree perineal laceration and there was especially no case of vaginal laceration in this group.

Collectively a resection of the rectum had been performed in 6 cases. One of these six women gave birth spontaneously, while in the remaining five cases a caesarean section was performed (Table 3). The woman who gave birth spontaneously suffered from a second-degree perineal laceration, but there was no injury of the rectum or the vaginal wall.

Literature describes that rectum resection due to endometriosis may lead to several complications, including rectovaginal fistula or anastomotic insufficiency [21]. A survey conducted by Remzi et al. described a significantly higher risk of sphincter injury in women who underwent an ileal pouch-anal anastomosis and afterwards delivered spontaneously compared to women who delivered via caesarean section [22].

Ravid et al. reported decreased long-term function in some women after ileal pouch-anal anastomosis because of ulcerative colitis after pregnancy, independently of the mode of delivery [23].

Although we found no such complications collectively, it is debatable if spontaneous delivery after rectum resection increases the risk of such complications and if abdominal delivery should be recommended in these cases.

A survey by Bulletti et al. found that spontaneous delivery can have a positive influence on dysmenorrhea and the recurrence of endometriosis. In this study, women that gave birth spontaneously had a longer pain-free interval than women on whom a caesarean section was performed [24]. However, as we did not investigate the postpartum period collectively, we cannot confirm or refute this finding.

A limitation of the present survey is the relatively small number of patients included. In our mind, further studies should be conducted, especially focusing on the mode of delivery after rectum resection and the influence on the mode of delivery on endometriosis associated symptoms such as dysmenorrhea.

## 5. Conclusion

Our study is the first description on delivery modes after operation for deeply infiltrating endometriosis. In our patient collective of women with spontaneous delivery, we did not have relevant laceration regarding an operation for DIE in history, even when a resection of the posterior vaginal wall or the rectum had been performed.

Concerning the small number of patients with resection of the rectum because of endometriosis and spontaneous delivery (*n* = 1), there is no conscientiousness for a recommendation.

Collectively, there was no higher risk of adverse pregnancy outcome after operation for DIE in history in women with spontaneous birth as well as in women with caesarean section, although women with lesions in compartments ENZIAN A and ENZIAN C as well as after rectum resection were more likely to have abdominal delivery.

## Figures and Tables

**Table 1:** Arterial umbilical blood pH.

	Min	Median	Max	*N*
Caesarean section	7.19	7.30	7.38	20
Spontaneous delivery	7.13	7.27	7.45	31

*Total*	*7.15*	*7.28*	*7.42*	*51*

**Table 2:** Patients with operation in compartment ENZIAN A.

	ENZIAN A	ENZIAN B	ENZIAN C	Open vagina	Delivery mode	Vaginal laceration
1	3	3	3	Yes	Secondary CS	
2	3	3	3		Primary CS	
3	2	0	2		Primary CS	
4	2	2	1	Yes	Primary CS	
5	2	2	0		Spontaneous delivery	No
6	2	2	0		Secondary CS	
7	2	0	0		Spontaneous delivery	No
8	1	2	2		Spontaneous delivery	No
9	1	1	1		Primary CS	
10	1	1	1		Primary CS	
11	1	0	0		Secondary CS	
12	1	0	0		Spontaneous delivery	No
13	1	2	0		Secondary CS	
14	1	1	0	Yes	Spontaneous delivery	No

**Table 3:** Patients with operation in compartment ENZIAN C.

	ENZIAN A	ENZIAN B	ENZIAN C	Open vagina	Rectum resection	Bladder resection	Delivery mode
1	3	3	3	Yes	Yes		Secondary CS
2	3	3	3		Yes		Primary CS
3	2	0	2		Yes		Primary CS
4	1	2	2		Yes		Spontaneous delivery
5	2	2	1	Yes	Yes	Yes	Primary CS
6	0	2	1				Spontaneous delivery
7	1	1	1		Yes		Primary CS
8	1	1	1				Primary CS

**Table 4:** Influence of DIE on the mode of delivery.

Severity of infiltration of the vagina and rectovaginal septum (ENZIAN A)	*p* = 0.020
Severity of infiltration rectum (ENZIAN C)	*p* = 0.031
Resection of the rectum	*p* = 0.029

## Additional Points

Women with histologically verified endometriosis do not have a higher risk of adverse pregnancy outcome but are more likely to have a caesarean section.

## References

[1] Bulun S. E. (2009). Endometriosis. *New England Journal of Medicine*.

[2] Bazot M., Lafont C., Rouzier R., Roseau G., Thomassin-Naggara I., Darai E. (2009). Diagnostic accuracy of physical examination, transvaginal sonography, rectal endoscopic sonography, and magnetic resonance imaging to diagnose deep infiltrating endometriosis. *Fertility and Sterility*.

[3] Brosens I., Brosens J. J., Fusi L., Al-Sabbagh M., Kuroda K., Benagiano G. (2012). Risks of adverse pregnancy outcome in endometriosis. *Fertility and Sterility*.

[4] Haas D., Wurm P., Schimetta W. (2014). Endometriosis patients in the postmenopausal period: pre- and postmenopausal factors influencing postmenopausal health. *BioMed Research International*.

[5] Haas D., Chvatal R., Reichert B. (2012). Endometriosis: a premenopausal disease? Age pattern in 42,079 patients with endometriosis. *Archives of Gynecology and Obstetrics*.

[6] Vigano P., Corti L., Berlanda N. (2015). Beyond infertility: obstetrical and postpartum complications associated with endometriosis and adenomyosis. *Fertility and Sterility*.

[7] O'Leary S. M. (2006). Ectopic decidualization causing massive postpartum intraperitoneal hemorrhage. *Obstetrics and Gynecology*.

[8] Costa A., Sartini A., Garibaldi S., Cencini M. (2014). Deep endometriosis induced spontaneous colon rectal perforation in pregnancy: laparoscopy is advanced tool to confirm diagnosis. *Case Reports in Obstetrics and Gynecology*.

[9] Pisanu A., Deplano D., Angioni S., Ambu R., Uccheddu A. (2010). Rectal perforation from endometriosis in pregnancy: case report and literature review. *World Journal of Gastroenterology*.

[10] Brosens I. A., De Sutter P., Hamerlynck T. (2007). Endometriosis is associated with a decreased risk of pre-eclampsia. *Human Reproduction*.

[11] Falconer H. (2013). Pregnancy outcomes in women with endometriosis. *Seminars in Reproductive Medicine*.

[12] Stephansson O., Kieler H., Granath F., Falconer H. (2009). Endometriosis, assisted reproduction technology, and risk of adverse pregnancy outcome. *Human Reproduction*.

[13] Conti N., Cevenini G., Vannuccini S. (2015). Women with endometriosis at first pregnancy have an increased risk of adverse obstetric outcome. *The Journal of Maternal-Fetal & Neonatal Medicine*.

[14] Lin H., Leng J.-H., Liu J.-T., Lang J.-H. (2015). Obstetric outcomes in chinese women with endometriosis: A Retrospective Cohort Study. *Chinese Medical Journal*.

[15] Vercellini P., Parazzini F., Pietropaolo G., Cipriani S., Frattaruolo M. P., Fedele L. (2012). Pregnancy outcome in women with peritoneal, ovarian and rectovaginal endometriosis: a retrospective cohort study. *BJOG: An International Journal of Obstetrics and Gynaecology*.

[16] Group EEGD

[17] Ulrich U., Buchweitz O., Greb R. (2013). Interdisciplinary S2k guidelines for the diagnosis and treatment of endometriosis. *Geburtshilfe Frauenheilkd*.

[18] Haas D., Oppelt P., Shebl O., Shamiyeh A., Schimetta W., Mayer R. (2013). Enzian classification: does it correlate with clinical symptoms and the rASRM score?. *Acta Obstetricia et Gynecologica Scandinavica*.

[19] Austria S. (2015). *Statistik der Natürlichen Bevölkerungsbewegung*.

[20] Menzlova E., Zahumensky J., Gürlich R., Kucera E. (2014). Rectal injury following delivery as a possible consequence of endometriosis of the rectovaginal septum. *International Journal of Gynecology and Obstetrics*.

[21] Klugsberger B., Shamiyeh A., Oppelt P., Jabkowski C., Schimetta W., Haas D. (2015). Clinical outcome after colonic resection in women with endometriosis. *BioMed Research International*.

[22] Remzi F. H., Gorgun E., Bast J. (2005). Vaginal delivery after ileal pouch-anal anastomosis: a word of caution. *Diseases of the Colon and Rectum*.

[23] Ravid A., Richard C. S., Spencer L. M. (2002). Pregnancy, delivery, and pouch function after ileal pouch-anal anastomosis for ulcerative colitis. *Diseases of the Colon and Rectum*.

[24] Bulletti C., Montini A., Setti P. L., Palagiano A., Ubaldi F., Borini A. (2010). Vaginal parturition decreases recurrence of endometriosis. *Fertility and Sterility*.

